# Maternal Macronutrient Consumption and the Developmental Origins of Metabolic Disease in the Offspring

**DOI:** 10.3390/ijms18071451

**Published:** 2017-07-06

**Authors:** Stephanie M. Kereliuk, Gabriel M. Brawerman, Vernon W. Dolinsky

**Affiliations:** 1 Department of Pharmacology & Therapeutics, University of Manitoba, Winnipeg, MB R3E 3P4, Canada; umkereli@myumanitoba.ca (S.M.K.); umbraweg@myumanitoba.ca (G.M.B.); 2 Diabetes Research Envisioned and Accomplished in Manitoba (DREAM) Research Theme of the Children’s Hospital Research Institute of Manitoba, University of Manitoba, Winnipeg, MB R3E 3P4, Canada

**Keywords:** developmental programming, metabolic disease, cardiovascular disease, high-fat diet, sucrose

## Abstract

Recent research aimed at understanding the rise in obesity and cardiometabolic disease in children suggests that suboptimal maternal nutrition conditions organ systems and physiological responses in the offspring contributing to disease development. Understanding the mechanisms by which the macronutrient composition of the maternal diet during pregnancy or lactation affects health outcomes in the offspring may lead to new maternal nutrition recommendations, disease prevention strategies and therapies that reduce the increasing incidence of cardiometabolic disease in children. Recent mechanistic animal model research has identified how excess fats and sugars in the maternal diet alter offspring glucose tolerance, insulin signaling and metabolism. Maternal nutrition appears to influence epigenetic alterations in the offspring and the programming of gene expression in key metabolic pathways. This review is focused on experimental studies in animal models that have investigated mechanisms of how maternal consumption of macronutrients affects cardiometabolic disease development in the offspring. Future research using “-omic” technologies is essential to elucidate the mechanisms of how altered maternal macronutrient consumption influences the development of disease in the offspring.

## 1. Introduction

Until recently obesity and type 2 diabetes were considered to be health problems of adult populations, yet since the 1980s the prevalence of obesity and diabetes in children has risen steadily. In addition, childhood overweight and obesity tracks into adulthood and is associated with a higher risk of chronic cardiometabolic diseases such as type 2 diabetes and cardiovascular disease [1]. Therefore, understanding the molecular and physiological mechanisms responsible for these epidemiological trends can better inform appropriate prevention strategies to reduce the immense health care burdens associated with these diseases. Emerging evidence suggests that suboptimal maternal nutrition has an influence on the health of the offspring during fetal development and infancy, as well as later on in childhood and into adulthood. Furthermore, the evidence suggests that adaptive responses to nutrition (both pre- and postnatally), mediated at the molecular level by alterations in gene expression, may predispose individuals to cardiometabolic diseases later in life [2]. In this review we highlight how suboptimal consumption of macronutrients during pregnancy influences cardiometabolic health outcomes in the offspring.

According to the Developmental Origins of Health and Disease (DOHaD) theory, the maternal intrauterine environment plays a key role in fetal development and conditions the offspring for risk of certain metabolic diseases as the offspring age. These disorders have been reported to include obesity, type 2 diabetes and cardiovascular disease [3,4,5,6]. David Barker was the first to provide evidence for this theory based on epidemiological studies of British children born in the 1920s. Barker identified associations between rates of ischemic heart and coronary artery disease mortality in adulthood in offspring exposed to undernutrition during gestation and born with low birth weights [5,7,8]. In addition to these studies, the Dutch famine cohort of 1944–1945 provided further evidence supporting the DOHaD theory. Briefly, a region of the Netherlands experienced reduced food availability during World War II, and after the war food availability returned to normal [9]. These studies found that children born during the famine had decreased fetal growth, reduced glucose tolerance, higher waist circumference, obesity, hyperlipidemia and an increased risk of type 2 diabetes, coronary artery disease, and mortality when compared with children born after the famine ended [9,10,11,12,13]. In contrast, correlations between intrauterine undernutrition and glucose intolerance, hyperlipidemia and cardiovascular disease in adulthood were not observed in the Leningrad Siege Study, where the population also suffered from a lack of food availability during and after the war [14,15]. The discordance between these two studies has been hypothesized to be due to the fact that the Leningrad population did not experience a period of sufficient food availability after the war, in contrast to the Netherlands population; therefore, offspring were exposed to fewer nutrients during and after birth [16]. Collectively, these studies suggest that undernutrition during pregnancy has serious repercussions for health outcomes early in the life of the offspring, as well as later on in adulthood. These studies also provided the foundation for the concept that the first 1000 days (from pregnancy to two years of age) are critical for a child’s development and future health [17].

While human studies are important to the field, it is difficult to control for confounding factors over a lengthy and expensive study period and genetic variability adds a high level of complexity. Therefore, rodent models with a common genetic background and carefully controlled dietary and activity conditions are useful for examining how altered macronutrient consumption during gestation or early postnatal life influences the development of obesity, insulin resistance and cardiovascular disease phenotypes in the offspring. On the other hand, there are significant limitations that need to be considered when using animals to model human pregnancy that can impact the generalizability of the findings. For example, the number of offspring per pregnancy, placentation, gestational length, parturition and different windows of fetal and neonatal cellular differentiation and organogenesis [18]. In addition, the normal macronutrient intake for rodents as a percent of total energy intake can be quite different from humans. Initial studies in animal models focused on how reductions in total caloric intake and protein restriction contributed to metabolic programming in the offspring, though other models of nutrient restriction are also used [19]. More recent research has expanded our understanding of how caloric excess from high saturated fats, high fructose/glucose, and high-fat/high-sucrose diets condition the offspring for the metabolic syndrome. It has also identified smoking, alcohol, levels of environmental toxins, and micronutrients during pregnancy as factors that influence the health of the offspring. Our review is focused on experimental studies in animal models that have investigated how suboptimal consumption of macronutrients affects the development of metabolic disorders in the offspring. In addition, we review studies of epigenetic mechanisms that could explain how maternal macronutrient consumption could affect offspring health.

## 2. Protein Restriction

Long-term preconception protein restriction (20%) has major negative effects on murine placental function and offspring growth and development, including cranial size [20]. The maternal low-protein diet model (in association with increased carbohydrate or fat) during gestation and lactation is one of the most extensively studied animal models of the effects of macronutrient deficiency on the offspring (Table 1). This model has been used in species ranging from sheep and pigs to rodents [19]. Feeding a low-protein diet (8% protein) during gestation and lactation is associated with growth restriction, increased blood pressure, fasting insulin and lipid levels and a generalized reduction in organ growth with brain and heart sparing effects in most of the rodent studies [21,22,23]. Maternal protein restriction affects the insulin sensitivity of the offspring. In a very well-designed study, Zambrano et al. [24] found that in rats, maternal protein restriction throughout pregnancy and lactation, as well as protein restriction only during lactation, induced improved insulin sensitivity in the male offspring, while protein restriction only during pregnancy and not lactation induced insulin resistance in the male offspring. Similar to the male offspring, protein restriction during pregnancy and lactation improved the insulin sensitivity of the female offspring, but protein restriction only during pregnancy did not impact the insulin sensitivity of the female offspring [24]. Chen et al. [25] also reported improved insulin sensitivity in the offspring of dams that were protein restricted throughout pregnancy and lactation, whereas the offspring that were protein restricted only during pregnancy and not lactation developed insulin resistance. The mechanism for these findings appeared to be increased insulin receptor signaling mediated by protein kinase C (PKC)-ζ in skeletal muscle in the offspring of dams that were protein restricted throughout pregnancy and lactation, whereas the expression of these proteins were reduced in the offspring of dams that were protein restricted only during pregnancy [25]. Maternal restriction of dietary protein throughout gestation and lactation in rats decreased hepatic triacylglycerol content in the male, but not female offspring [26]. This effect could be due to increased fatty acid transport into the mitochondrial matrix or alterations in triacylglycerol biosynthesis [27]. Maternal protein restriction in pigs was shown to reduce the lean mass and increase fat mass in six-month-old offspring, with a tendency for reduced number of muscle myofibers associated with reduced expression of insulin-like growth factor 2 (*IGF2*) mRNA [28]. Consistent with protein restriction having an effect on lipid metabolism, maternal protein restriction resulted in smaller adipocytes in the fat tissues of offspring exposed to protein-restriction during pregnancy and lactation [29]. Despite their smaller size, the insulin sensitivity of these adipocytes was impaired in association with reduced protein expression of key insulin signaling molecules (e.g., Akt) [29]. In the male offspring from a sheep model, protein restriction only during pregnancy (but adequate early postnatal nutrition) exhibited cardiac hypertrophy and altered cardiac function, while high blood pressure was observed when protein was restricted only during early postnatal life [30]. Using the apolipoprotein E (ApoE) knock-out mouse model that is sensitive to the development of atherosclerosis, Blackmore et al. [31] showed that maternal protein restriction increased the atherosclerotic plaque area in the aorta of six-month-old male offspring. Moreover, maternal protein restriction of the ApoE knock-out mice was associated with elevated low density lipoprotein (LDL) cholesterol levels and fasting insulin levels as well as increased 3-hydroxy-3-methyl-glutaryl-coenzyme A (HMG-CoA) reductase expression in offspring livers [31]. Of note, protein restriction during lactation prevented catch-up growth in the offspring. Thus, it appears that cardiometabolic health outcomes in the offspring were sensitive to both the length and timing of maternal protein restriction and the postpartum growth trajectory throughout the postnatal period. Both rapid catch-up growth and weight gain appear to augment metabolic alterations in end organs such as the liver [32], while the absence of postnatal catch-up growth improved whole body insulin sensitivity in the offspring [33].

Epigenetic modifications are critical during periods of development and are influenced by environmental exposures that involve changes in gene expression without changes in the DNA sequence. These modifications include non-coding RNA, DNA methylation, histone methylation and acetylation. Epigenetic mechanisms that regulate gene expression such as DNA or histone methylation can be sensitive to nutrition. Since many of these epigenetic marks are established early in development and can persist for a lengthy period of time, epigenetic modifications are widely hypothesized to be an overarching mechanism linking maternal nutrition to metabolic health phenotypes in the offspring [34]. The strongest evidence linking epigenetic mechanisms to the effects of maternal nutrition on the health of their offspring comes from research on maternal undernutrition. For example, a recent genome-wide DNA methylation analysis of same-sex siblings in the Dutch Hunger Winter Famine cohort revealed a pattern of malnutrition-associated differentially methylated regions that were highly represented in genes involved in developmental and metabolic regulation [35]. In particular, maternal protein restriction reduces the supply of methyl groups from glycine when fetal demand is high. Consistent with this concept, a reduction in overall maternal calories during pregnancy induced hypomethylation of promoters in the liver tissue of the offspring that resulted in the increased expression of genes involved in fatty acid oxidation (e.g., peroxisome proliferator-activated receptor (*PPAR*)*-α* and *PPAR-γ* Coactivator (*PGC*)*-1α* and reduced expression of genes involved in lipid synthesis (e.g., *sterol response element binding protein-1c* and *diacylglycerol acyltransferase-1*) [36]. Maternal protein restriction during pregnancy induced hypomethylation of both the glucocorticoid receptor and the *PPAR-α* promoters in the livers of the offspring that conditioned alterations in the expression of their target genes and metabolic processes under the control of these transcription factors [37,38]. In the islets of the offspring, maternal protein restriction altered histone methylation and increased DNA methylation of the hepatocyte nuclear factor 4*α* (*HNF4α*) promoter that ultimately resulted in reduced *HNF4α* expression [39]. Supplementation of a low-protein diet with glycine and folate can prevent epigenetic and phenotypic effects on the offspring [40,41,42]. While studies on protein restriction were at the forefront of the studies on the developmental origins of disease, the larger issue in our modern society is the addition of simple sugars and saturated fats, which will be of emphasis in the following review.

## 3. Carbohydrates: Maternal Diets High in Simple Sugars and the Influence on Offspring

While people in the developing world are faced with undernutrition, improving living standards have increased the consumption of calorie-dense foods and consequently, much of the world’s populace is faced with over nutrition, characterized by excess levels of macronutrients such as refined sugars and saturated fats. Evidence is emerging that excess levels of these macronutrients in the maternal diet can contribute to hyperglycemia during fetal development and are associated with an increased prevalence of type 2 diabetes in adulthood [43]. Furthermore, maternal diets containing high levels of saturated fats or refined sugars appear to be a factor in the pathogenesis of obesity and metabolic syndrome in the offspring [44,45]. Based on the Healthy Start study, maternal Body Mass Index and over nutrition (high-fat diet composed of more than 30% of calories from fat and more than 12% of the fat calories from saturated fats) can lead to obesity, insulin resistance and impaired glucose utilization in the mothers. These maternal metabolic conditions influence increased fetal growth, birth weights, and the development of obesity and type 2 diabetes later in the life of their offspring [46,47].

It is widely appreciated that the consumption of foods and beverages that are sweetened with simple sugars are contributing to the rising incidence of obesity and diabetes in the developed world as well as in developing nations as their populations increase the amount of refined sugars in their diets. Not only do diets high in refined sugars have a major effect on health, but evidence is emerging that consumption of simple sugars during pregnancy have an effect on health outcomes in their offspring. In this section, we describe the influence of maternal sugar consumption on obesity, insulin resistance, and cardiovascular disease in the offspring (summarized in Table 2).

Several animal studies have found that maternal diets consisting of 75% simple carbohydrates (dextrose and maltodextrin) led to a significantly higher body weight gain in both mothers and offspring when compared against mothers eating a diet composed of 35% dextrose and maltodextrin [48,49,50,51,52,53]. These studies used an overfeeding model of the different diets through total enteral nutrition. Sprague–Dawley rat mothers that consumed a high carbohydrate diet through intragastric infusion for three weeks had increased body weight gain, increased adipose tissue, and increased levels of serum insulin and leptin, consistent with an insulin resistant state [48]. After weaning, the offspring were randomly assigned to either a control or a high-fat diet (45% fat calories). Only the male rat offspring from the dams that consumed the high carbohydrate diet exhibited increased body weights and higher percent body fat when compared to the offspring from dams consuming the lower carbohydrate control diet [48]. Another study feeding the same dextrose and maltodextrin diet compositions to the dams found that the higher carbohydrate diet-induced an upregulation of several lipogenic and adipogenic genes in the white adipose tissue of the male rat offspring at 21 days of age, which can prime the offspring for the development of obesity [53]. Furthermore, they suggested that in the offspring of dams receiving the higher carbohydrate diet, these effects were due to changes in DNA methylation in regulatory elements of genes required for development. For example, *PPAR-γ*, *CCAAT enhancer binding protein-α* and *-β* expression were all increased, indicating an adipogenic gene expression profile [53]. Interestingly, these studies have shown that increased obesity in the offspring may be programmed in utero depending on the maternal diet consumed and that nutrients, in this case, carbohydrates, can indeed change DNA methylation leading to changes in gene expression responsible for adipogenesis in the white adipose tissue. This would indicate the importance of the white adipose tissue to the organism as a whole in determining the potential for obesity and the future comorbidities associated with it. Furthermore, it is important to note, that these studies, in particular, focused on the male offspring without reporting findings for the female offspring. This is an important caveat since the offspring could respond differently to the fetal programming effects of the maternal diet composition due to sex differences. Fortunately, sex are not overlooked as frequently since more studies are reporting data from male and female offspring. In summary, these studies suggest that maternal obesity caused by a high carbohydrate diet can have a significant effect on the body weight of their offspring.

In addition to elevated adiposity, several studies have found that insulin sensitivity is also affected in the offspring of dams fed a high-carbohydrate diet. In fact, male rat offspring of dams that consumed a high-sugar diet had increased serum insulin, leptin, and resistin levels at 21 days of age, which is an early indication of insulin resistance [49]. Another study that followed the male offspring to 130 days of age, found that offspring of both lean (35% dextrose and maltodextrin) and obese (75% dextrose and maltodextrin) dams had significantly increased levels of serum glucose, triglycerides, insulin, and leptin. However, when the male offspring of high carbohydrate-fed dams were fed a postnatal high-fat diet, they had higher serum insulin and leptin levels than the offspring of the lower carbohydrate-fed dams on the same postnatal diet [48]. The increased obesity in the offspring of high carbohydrate-fed dams was linked with a 3-fold increase in fasting levels of insulin. Upon performing an oral glucose tolerance test to measure their insulin response, these offspring were clearly insulin intolerant since their glucose levels did not decrease over time [48]. In summary, these findings suggest that a maternal diet composed of high levels of simple sugars can lead to impaired glucose sensitivity and insulin intolerance in the offspring.

Furthermore, some studies have found a link between a maternal diet rich in simple carbohydrates and alterations in the expression of mitochondrial function markers, which can predispose the offspring to obesity and insulin resistance. In fact, many hepatic mitochondrial function markers were found to be downregulated in the rat offspring of dams that were intragastrically fed a 75% dextrose and maltodextrin diet [52]. For example, *SIRT3*, electron transport chain complexes II, III and *ATPase*, and *PGC-1α* mRNA were all reduced in the livers of the offspring of the dams that consumed the high carbohydrate diet during pregnancy [52]. Another study found that mitochondrial fusion and fission, which are required for proper mitochondrial function, were highly affected in the offspring of dams that consumed the high carbohydrate diet during pregnancy [51]. More importantly, an imbalance between these two processes can lead to obesity and insulin resistance [51]. This study found that some key regulators of mitochondrial fusion and fission in the liver were reduced at both 35 and 130 days of age in the rat offspring which included *PARL*, *optic atrophy-1*, *mitofusin-1* and *-2*, *fission-1*, and *nuclear respiratory factor-1* [51]. Therefore, a maternal diet high in simple carbohydrates can have significant repercussions on mitochondrial health in the offspring, which could eventually lead to obesity and insulin resistance.

Maternal diets throughout pregnancy that are high in fructose have similar damaging effects on maternal and offspring health as the high dextrose/maltodextrin enriched diets. In rats, a 50% fructose diet during pregnancy was sufficient to induce maternal hyperglycemia, hyperlipidemia and elevated hepatic gluconeogenesis [54]. Newborn pups from these pregnancies were hyperglycemic [54]. In a separate study, the offspring of dams that consumed a 60% fructose diet also had hyperinsulinemia and elevated serum lipids [55]. This was associated with alterations in several genes involved in lipid metabolism, including increased *acetyl-CoA carboxylase-2*, *carnitine palmitoyl transferase* (*CPT*)*-1α* and reduced expression of *PPAR-α* and *PGC-1α* [55]. However, these studies used levels of fructose that markedly exceed the fructose consumption in human diets. More recent studies have utilized more relevant levels of fructose. Consumption of 10% fructose by pregnant rats altered both maternal and fetal leptin signaling suggesting a potential influence of fructose on body weight [57]. Increased body weight and food consumption was observed in the two-month-old male rat offspring of dams that consumed 10% fructose during pregnancy and lactation [58]. This was likely a consequence of reduced hypothalamic responsiveness to leptin and reduced expression of anorexigenic peptides [58]. More recently, using a 20% fructose diet during pregnancy, Clayton et al. [59] reported maternal fructose consumption induced significant alterations in liver lipid metabolism in neonatal offspring. While maternal fructose consumption did not induce obesity in either male or female neonatal offspring, liver triacylglycerol levels were elevated and the expression of molecular markers of endoplasmic reticulum (ER) stress were induced [59]. Interestingly, a large subset of genes involved in free fatty acid metabolism that were suppressed in the liver of male offspring from fructose-fed dams were unaffected in the livers of the female offspring. These genes included *acyl-CoA cholesterol acyltransferase-1*, *long-chain fatty acyl-CoA synthase-4*, *acyl-CoA dehydrogenase-10* and *CPT-1α* [59].

Finally, there is some evidence that a maternal diet high in simple sugars can condition the offspring for the development of kidney dysfunction and high blood pressure, leading to cardiovascular disease. An older study showed the effects of hyperglycemia on kidney development in rats [61]. The authors demonstrated that exposure to high glucose levels in utero resulted in lower numbers of nephrons, which can evolve into renal failure in adulthood. They found that the number of nephrons in the offspring from a streptozotocin-treated dam was significantly reduced when compared to control offspring due to high levels of glucose in the fetal environment [61]. Another study found that a maternal diet consisting of 60% high fructose led to the programming of hypertension in the adult male offspring of Sprague–Dawley rats. Additionally, they found some genes that would normally regulate blood pressure to be differentially expressed in the kidneys, thus resulting in increased blood pressure in the offspring [56]. Consistent with this finding, the consumption of a high-carbohydrate diet (more than 40% of energy) by mothers during their pregnancy was associated with elevated systolic blood pressure in their four-year-old children [62]. A different study found that the male rat offspring of dams that were fed a 20% sucrose solution during pregnancy, exhibited an increase in angiotensin II in the bloodstream which caused vasoconstriction in the aorta and mesenteric arteries [60]. The authors argued that this increase in angiotensin II can lead to higher blood pressure and the risk of hypertension in the aged offspring [56]. In summary, kidney dysfunction can result in higher blood pressure which, if left untreated, can turn into a more serious cardiac condition and eventually heart failure [63]. Therefore, maintenance of normoglycemia during pregnancy is essential in order to prevent fetal exposure to potentially dangerous levels of sugars, which can have detrimental effects on the health of the offspring [61]. However, there seems to be a lack in the literature about maternal diets high in simple carbohydrates during pregnancy and the effects on heart size, structure, and function in the offspring. To date most of the experimental research has involved combining a high-fat diet and some percentage of carbohydrate in the maternal diet during pregnancy, thus making it impossible to differentiate if the effects observed are due to the high fat or the sugar. It will be important for research groups to implement studies where different concentrations and types of carbohydrates are used in the maternal diets, to further elucidate what levels of maternal carbohydrate consumption as well as what types of carbohydrates present the worst outcomes on the development of cardiovascular disease in the offspring and any programming that may be taking place at specific stages of fetal development.

## 4. Fatty Acids: Maternal Diets High in Saturated Fat Diets and Their Influence on the Offspring

Maternal high-fat diet feeding has been used to investigate the effects of saturated fats, excessive gestational weight gain and maternal obesity on the offspring. As a result, high-fat feeding during pregnancy has several effects on maternal metabolism and body composition, including increased adiposity, insulin resistance, hyperinsulinemia and increased circulating lipids (Table 3). However, increasing fat in the diet alters the composition of other macronutrients in the diet, often reducing the carbohydrate and protein composition to match the increase fatty acid. Additionally, many of these studies have used fat compositions that vary (from 20% up to 60% kcal using different sources of fat (lard, corn oil, etc.) to investigate the effects of fat in the maternal diet on the offspring. The differences in other macronutrients with high and low-fat diets are also indicated in Table 3 and Table 4 under % Fat. Altering these additional macronutrients allow for investigators to study the implications of different macronutrient combinations as well as the types of fats used in the maternal diets on cardiometabolic health in offspring.

Using a maternal diet supplemented with 20% *w*/*w* animal lard containing oleic, palmitic and stearic acids as the highest composition of the fats in the diet, Taylor et al. demonstrated that the placenta and fetal offspring body weights were lower in lard-fed dams on embryonic day 20 [64]. The effects of the maternal lard-based high-fat diet on the development of metabolic syndrome were observed in the offspring as they aged. Female offspring were fed a postnatal chow diet up to one year of age and exhibited increased body weight, insulin resistance, elevated plasma leptin, fasting glucose, and insulin levels [66,68], although the triglyceride and cholesterol levels were unchanged. At three months of age, lower mitochondrial copy number and a 5-fold lower mRNA expression of the mitochondrial genome were observed in the liver and kidney tissues of the offspring of high-fat fed dams when compared to controls [66]. At nine months of age, impaired glucose-stimulated insulin secretion was apparent in the offspring of high-fat fed dams and this was associated with an impaired ex vivo glucose-stimulated insulin secretion without alterations in basal insulin release [66]. Islet insulin content was lower in offspring from high-fat-fed dams and electron micrographs showed that pancreatic islets had enlarged insulin secretory granules without a change in islet architecture [66].

Several reports using a similar maternal high-fat feeding of a lard-enriched diet demonstrated that adult rat offspring exhibited vascular endothelial dysfunction, increased arterial blood pressure, and fatty acid levels [2,64,68]. While one study did not observe alterations in plasma lipids [66], another showed that in one-year-old chow-fed female rat offspring, the maternal high-fat diet elevated plasma triacylglycerol and reduced total and high density lipoprotein (HDL) cholesterol [68]. Interestingly, when the offspring of high-fat-fed dams were weaned and then randomly assigned to a chow diet or the same lard-based high-fat diet the dams received, the impairments of cardiovascular function observed with post-weaning chow diet, were not observed in the offspring fed the post-weaning high-fat diet. For example, matching the maternal high-fat diet with the post-weaning high-fat diet prevented the development of impaired acetylcholine-induced endothelium-dependent relaxation, and reduced plasma triacylglycerol and glucose levels [2]. These findings suggest that a high content of long-chain saturated and monounsaturated fats in the maternal diet negatively affected the cardiovascular health of the offspring, especially when the fat content of the offspring diet did not match the maternal fat content.

Several other studies have used a protocol that involves feeding a high-fat diet (60 kcal% from fat, 20 kcal% from protein, 20 kcal% from carbohydrate) to study the effect of maternal overnutrition during the gestational period and not the pre-existing effect of maternal obesity. The fetal offspring of high-fat-fed dams exhibited a decreased, but non-significant trend in fetal body weight. Fetal body fat content was not different at E18.5, but body fat content as a percentage of total body mass was elevated compared to control offspring [65]. Placental triacylglycerol (TG) levels were increased in E18.5 offspring of the high-fat fed dams and a corresponding increase in fetal serum free fatty acids (FFA) was observed when compared to controls, suggesting that the maternal high-fat diet-induced increases in placental lipid metabolism genes (*LPL*, *CD36*, *VLDLr*, *FABP3*, etc.) that facilitated TG hydrolysis and fatty acid (FA) uptake/transport into the fetal circulation [65].

The quantity and quality of fat influence the development of cardiovascular disease, insulin resistance, and type 2 diabetes development. Alterations in the fatty acid profile of diets can affect insulin sensitivity. The general consensus indicates that the more unsaturated the fat, the less harmful it is in the maternal diet. Classes of unsaturated fats are also important to consider for disease development, as the omega-3 polyunsaturated fats (PUFA) are reportedly beneficial to insulin action, while omega-6 PUFA negatively impacted glucose homeostasis and insulin sensitivity [71]. One paper has examined how a diet high in omega-6 PUFA started four weeks before mating and continued only during gestation affects the chow-fed offspring at three months of age. The offspring exposed to the PUFA diet had similar body weight as the control group but had increased abdominal and total body fat [70]. While glucose tolerance and whole body insulin sensitivity were similar, hepatic insulin sensitivity was reduced in the offspring of PUFA dams in association with reduced insulin receptor and insulin receptor substrate-1 expression and elevated hepatic triacylglycerol levels [70]. Moreover, supplementing rat diets with oxidized fish oil containing oxidized PUFA had adverse effects on maternal insulin sensitivity and survival of newborn offspring, and also increased the insulin resistance of three-week-old offspring [72].

The suckling period is also a very critical window in which maternal high-fat diet programs body weight in the offspring. Khan et al. [69] cross-fostered offspring from a lard-based high fat-fed dam to a chow fed dam and the offspring from the chow-fed dams to the high-fat-fed dam. Interestingly, while body weight was similar in all groups and sexes at 6 months of age, all groups of male offspring exposed to high fat only during pregnancy, only during lactation, as well as during both pregnancy and lactation developed increased intra-abdominal fat accumulation [69]. In the female offspring, adiposity was only increased when the offspring from chow-fed dams were cross-fostered to the high-fat-fed dam. The male and female offspring exposed to high fat during pregnancy and lactation as well as during only pregnancy or only during lactation all exhibited hypertension and impaired acetylcholine-dependent arterial relaxation [69]. In a similar study, dams were fed normal chow (13.5% kcal from fat) or a high-fat diet (60% kcal from fat) during pregnancy and the offspring were cross-fostered on postnatal day one. Male and female pups fostered to high-fat-fed dams were heavier than those fostered to chow fed dams beginning on P7, which persisted throughout lactation (until P21) [67]. At P21 both male and female pups cross-fostered to the high-fat diet dams, had greater adiposity (visceral and subcutaneous) and plasma leptin levels [67]. At nine weeks of age, the male offspring cross-fostered to high-fat-fed dams, had elevated body weights and increased subcutaneous fat, though these parameters were not different in the corresponding female offspring [67].

Overall these studies investigating the developmental programming of offspring from maternal high-fat diet both during pregnancy and lactation seldom investigated the molecular mechanisms/gene expression (Table 3). Future studies comparing the direct effects of different types of fatty acids on the offspring would be valuable, especially if they are able to evaluate how fat specifically during gestation or during the suckling period influences the health of the offspring. More studies that examine how fats in the maternal diet influence epigenetic programming, gene expression and molecular signaling pathways in organ systems of the offspring are needed.

## 5. Maternal Diets Containing Combinations of High Saturated Fats and Simple Carbohydrates and Their Effect on Offspring Health

While maternal diets high in glucose/fructose (Table 2) or high in saturated fats (Table 3) have detrimental effects on offspring health, the consumption of refined foods in the modern diet usually involves the combination of both simple sugars and high levels of fat. Increasing numbers of women are consuming diets high in saturated fats as well as simple sugars that contribute to excess weight gain during their pregnancy [73]. As a result, research that investigates the effects of maternal macronutrient excess during pregnancy on the health outcomes of the offspring increasingly utilizes diets that are high in a combination of saturated fats and sucrose (summarized in Table 4).

Maternal diets as low in fat content as 11% kcal fat and 43% kcal of simple sugars have an effect on the development of obesity in the offspring. In their study, Nivoit et al. [86] showed that this maternal diet composition induced hyperphagia, elevated body weight and adiposity in both male and female offspring fed a post-weaning chow diet up to 12 months of age. As early as three months of age, glucose intolerance was apparent in the offspring of high fat and sugar fed dams [86]. Insulin resistance assessed using euglycemic-hyperinsulinemic clamps was observed in nine-month-old females and 11-month-old male offspring of high-fat- and –sugar-fed dams [86].

Using a slightly higher fat content (16% kcal) and a lower quantity of simple sugars (33% kcal) in the maternal diet, several studies using mice have evaluated the effect that this maternal diet during pregnancy and lactation have on the development of obesity, hepatic steatosis and pancreas dysfunction in the offspring. The offspring of high-fat- and -sugar-fed dams were heavier [67,87,89]. This was associated with elevated hepatic steatosis and increased alanine aminotransferase and hepatic fibrosis as these offspring aged [87,88]. Yu et al. [90] observed global hypermethylation of genes in the liver tissues of offspring from dams fed a diet containing 16% fat from lard. A number of these genes involved in fat homeostasis such as *liver X receptor* (*LXR*)*-α* and *PPAR-γ* were hypermethylated and a corresponding reduction in mRNA and nuclear protein content was observed [90]. Using the non-human primate Japanese macaque model of maternal high-fat (35% kcal) diet, fetal liver triacylglycerol levels were increased, histone H3K14 was hyperacetylated and the expression of histone deacetylase-1 protein was reduced [91]. In addition, increased Kupffer cell number and reduced natural killer T-cell numbers were observed [87]. Offspring of the high-fat and sugar dams had increased levels of hepatic cytokine production (e.g., interleukin (IL)-6, tumor necrosis factor (TNF)-α and tumor growth factor (TGF)-β) and greater liver injury when they were weaned on to the same high fat and high simple carbohydrate diet [87]. Interestingly, when the offspring of the lean control dams were cross-fostered to mothers that were consuming the high-fat and high-carbohydrate diet during lactation, increased body weight, hepatic steatosis, IL-6 and TNF-α production were observed [88]. However, a maternal diet high in fat (58% kcal) caused multiple changes in the expression of genes involved in inflammation, glucose and cholesterol metabolism in the livers from nine-week-old male mouse offspring without altering DNA methylation in these tissues [92]. These findings suggested that the maternal diet composition during lactation was very important in the programming of liver function in the offspring. In addition to excess abdominal fat accumulation and hepatic steatosis, the same maternal high fat and carbohydrate feeding protocol during pregnancy induced lipid accumulation in the whole pancreas of the offspring [89]. This was associated with an increase in stress-related changes such as the unfolded protein response in the pancreatic ER [89]. Pancreatic gene expression of downstream regulators of the unfolded protein response and autophagy-related proteins in the offspring were altered by the high-fat and high-sugar diet during pregnancy. For example, total and phosphorylated *Eif2α* were decreased and X-box binding protein 1 (XBP1) expression was increased in the mouse pancreas of these offspring at six months of age [89]. While a limitation of this study is that the researchers did not directly examine the accumulation of fat within the pancreatic islets specifically, the development of fatty pancreas is associated with β-cell dysfunction [93] as well as greater pancreatic inflammation and fibrosis [94], which can have negative effects on the insulin secretion capacity of the β-cells of the pancreas.

Similar to the studies described above, feeding female mice a diet containing 28% kcal fat and 55% kcal simple carbohydrates prior to pregnancy, throughout pregnancy and lactation increased obesity, hepatic steatosis and elevated insulin resistance and cardiovascular disorders in offspring weaned onto a chow diet compared to the chow-fed offspring of chow-fed dams. The maternal consumption of this diet high in fat and simple carbohydrates was also associated with increased body weight gain in the offspring at three months of age or older [78,79], but not at two months of age [80,82]. The increased body weight was primarily due to increased adipose tissue [78]. These offspring were less active and consumed more food [78,79]. The mechanism appeared to involve modification in hypothalamic systems regulating appetite, including reduced *STAT3* phosphorylation in the arcuate nucleus and reduced *AgRP* expression in the periventricular hypothalamus that appeared to be related to a prolonged neonatal leptin surge and the development of leptin resistance [79]. As previously reviewed, protein restriction (8% protein in the diet) from conception until weaning affected DNA methylation of ribosomal DNA sequences in mice and similar effects were found with maternal high-fat (20% kcal from animal lard) and simple sugar (10% kcal) diet feeding [79]. Interestingly, adipose tissue cytokine and chemokine signaling were upregulated in the two-month-old offspring of high-fat and simple sugar-fed dams, which preceded obesity development [85]. The reduction in miR-706, which regulates the translation of IL-33 and the calcium/calmodulin kinase-1d, in the adipose tissues of these offspring appeared to be responsible [85]. Notably, reduced tibialis anterior muscle mass was observed [78], suggesting that a reduction in metabolically active lean mass could contribute to excess adiposity. Although myoblasts and adipocytes originate from the same mesenchymal precursor cells, no studies have investigated whether fetal exposure to the maternal high-fat and simple sugar diet shifts cells from a muscle cell lineage to an adipocyte lineage. However, differences in muscle fiber density were not observed in the offspring of high-fat and simple sugar-fed dams, compared to the offspring of chow-fed dams [85]. Nonetheless, reduced activity of mitochondrial electron transport chain complexes 2 and 3 were observed [81], suggesting that the muscle tissue of these offspring were less metabolically active. Mitochondrial function of liver tissues from these offspring was also affected. Mitochondrial cytochrome c expression was reduced, while mitochondrial electron transport chain complexes 1 and 2 expression were increased, suggesting the presence of mitochondrial dysfunction that could contribute to the observed hepatic steatosis [83]. Fasting plasma glucose was reported in the offspring of high-fat- and carbohydrate-fed dams even in the presence of high levels of plasma insulin, suggesting marked insulin resistance [78]. Sex differences were observed in insulin signaling expression in offspring vastus lateralis muscle tissue. In male offspring, Akt2 and PKC-ζ protein expression were increased, but in female offspring phosphorylation of Akt (Ser473) was reduced and the expression of the catalytic subunit of phosphatidylinositol 3-kinase (PI3K) was also reduced [81]. Adipose tissue insulin sensitivity was also reduced at 8 weeks of age, which precedes the onset of obesity in the offspring of high-fat- and sugar-fed dams [82]. Consistent with this, reduced insulin receptor, insulin receptor substrate-1, PI3K, and Akt were observed in the adipose tissues [82]. Therefore, the influence of saturated fat and carbohydrate excess in the maternal diet appeared to have a significant effect on adipose and muscle tissues in the offspring that influenced their insulin sensitivity.

Exposure to the same 28% kcal fat and 55% kcal carbohydrate diet throughout gestation and lactation also predisposes the offspring to cardiovascular diseases. Endothelial dysfunction and hypertension were observed in the three-month-old offspring of high-fat- and carbohydrate-fed dams [78]. In three-month-old offspring, cardiac hypertrophy was reported [84], although cardiac hypertrophy was also apparent in two-month-old offspring, before the onset of obesity [80]. However, neither of these studies measured blood pressure in the mice, which causes an adaptive increase in the size of the heart. By three months of age, systolic and diastolic dysfunction was apparent in the isolated Langendorff perfused hearts from the offspring of high-fat- and carbohydrate-fed dams [84]. Several molecular markers of cardiac hypertrophy were also increased in the heart tissues from two-month-old offspring of high-fat- and carbohydrate-fed dams, including brain natriuretic peptide, myosin heavy chain and miR-133a [80]. Furthermore, expression of a cardiac fetal gene program was observed in the three-month-old offspring [84]. In addition, increased growth factor signaling, as suggested by increased phosphorylation of Akt1, and expression of extracellular-signal regulated kinases (ERK) 1/2 and the mechanistic target of rapamycin (mTOR) was observed, which could be related to hyperinsulinemia in these offspring [80]. Moreover, oxidative stress was also observed that was associated with reduced superoxide dismutase expression and increased levels of the lipid peroxide, 4-hydroxy-2-nonenal in the hearts [80]. While it is possible that the development of cardiovascular disease in these offspring could be secondary to effects of the maternal high-fat and simple sugar diet on other organ systems in the offspring that are reviewed above, the early-onset of many of these conditions that preceded obesity development in the rodent offspring suggests that maternal high-fat and simple sugar diets do specifically condition the cardiovascular system of the offspring for cardiovascular disorders.

Several groups have fed a 45% kcal high-fat and 35% kcal sucrose (HFS) diet to pregnant rats that induced maternal obesity. Howie et al. [74] showed that the newborn offspring of dams that were fed a HFS diet during pregnancy had a lower body weight but experienced rapid catch-up growth and by five months of age, offspring fed post-weaning chow or HFS diets were more obese, with elevated adiposity. Interestingly, this study showed that a lifetime of maternal HFS diet had the same effect on the offspring as feeding a HFS diet exclusively during pregnancy and lactation. A maternal HFS diet during pregnancy and lactation contributed to the development of hepatic steatosis in the rat offspring of HFS-fed dams at 15, 20 and 30 weeks of age [75,76]. At 15 weeks of age, impaired hepatic mitochondrial metabolism and increased hepatic lipogenesis were observed in the offspring of HFS-fed dams [76]. This involved the upregulation of hepatic genes involved in lipogenesis, oxidative stress resistance, and inflammation. Interestingly, altered liver development was apparent very early in life in the offspring of HFS-fed dams as the liver failed to undergo catch-up growth. Using flow cytometry to measure cell cycle dynamics in the liver cells from the two day-old newborn offspring of HFS-fed dams, inhibition of the transition from G_1_ to S-phase was observed, indicating reduced cell proliferation early in postnatal life [75]. However, in liver cells from postnatal day 27 offspring, the cells from the offspring of both groups of dams appeared quiescent (G_0_/G_1_) [75]. The expression of genes that encode cell cycle promoting proteins (*PCNA*, *cyclin A2*, *cyclin F*) was reduced and the expression of cell cycle inhibitory proteins (*p21^CIP/WAF1^*, also known as *cyclin-dependent kinase inhibitor 1*) were upregulated in liver cells from the two day-old offspring of HFS-fed dams [75]. Postnatal day 27 offspring had no changes in gene expression. These findings support the hypothesis that altered cell cycle dynamics in early postnatal life are associated with hepatic growth impairment and altered transcriptional regulation of cell cycle genes. Mechanistically, hypomethylation of the cell cycle inhibitor gene *Cdkn1α* corresponded to increased Cdkn1α expression in the livers of postnatal day 2 offspring from HFS-fed dams [75].

In addition to maternal obesity, our group has observed that starting the HFS diet six weeks prior to mating and continuing throughout pregnancy caused mid-gestational glucose intolerance, hyperinsulinemia and fasting hyperglycemia in rats, that fits the clinical criteria for gestational diabetes mellitus [45]. Newborn offspring of HFS dams were larger than the newborn offspring of lean dams [45]. At weaning, the offspring were randomly assigned to either a low-fat (10% fat) diet or matched to the maternal HFS diet. The offspring of the HFS-fed dams that consumed a post-weaning low-fat diet gained more weight than the offspring of the control dams and post-weaning HFS feeding had an additive effect on the development of obesity in the offspring of HFS-fed dams [45]. This corresponded to elevated adiposity and hepatic steatosis that appeared to contribute to marked insulin resistance in the 16-week-old offspring of HFS-fed dams [45]. In order to understand the mechanisms responsible, metabolomic profiling of tissues was utilized. Metabolomic analysis of liver tissue revealed increased diacylglycerol accumulation, which is a lipotoxic lipid and reduced phosphatidylethanolamine synthesis, which is an important membrane phospholipid [45]. Gene expression analysis of liver revealed a novel mechanism that suggested that reduced cytidine triphosphate phosphoethanolamine cytidylyltransferase expression (the rate limiting enzyme for phosphatidylethanolamine synthesis) reduced the utilization of diacylglycerols for phosphatidylethanolamine synthesis and ultimately resulted in diacylglycerol and triacylglycerol accumulation in the liver [45]. Metabolomic analysis of muscle tissue from the offspring of HFS-fed dams also revealed an accumulation of diacylglycerols [95]. This was associated with activation of PKC-δ, which is a mediator of muscle insulin resistance [95]. PKC-δ inhibited the expression of miR-133a in the muscle tissue of offspring from HFS-fed dams, which is a microRNA that suppresses the expression of the mitophagy and cell death modulating protein, Nix, thereby linking maternal HFS diet to the conditioning of the offspring to mitochondrial dysfunction [95]. A separate study observed that in the soleus muscles of five-month-old male offspring of HFS-fed dams, electron transport chain complex 1 and complex 3 activities were reduced and genes encoding the electron transport chain complex subunits were also reduced [96]. It appeared that the downregulation of the mitochondrial transcription factors, nuclear respiratory factor-1, and mitochondrial transcription factor A, that control the expression of mitochondrial genes could be responsible [96]. Since inflammation is associated with the development of insulin resistance, we investigated the immune reactivity of spleen cells isolated from the offspring of HFS-fed dams. In response to toll-like receptor activation, spleen cells from both neonatal and 16-week-old offspring of HFS dams experienced sustained stimulation of IL-1β and IL-10 production [77]. Therefore, the maternal consumption of a diet high in saturated fat and sucrose, primed the offspring for a chronic low-grade inflammation, obesity, lipotoxic conditions and mitochondrial dysfunction in the offspring in a manner that was independent of postnatal nutrition. Moreover, changes in food intake were not observed in the offspring characterized in these studies. However, no studies have directly compared maternal feeding of high saturated fat or high glucose/fructose diets to diets that contain combinations of high levels of saturated fats and simple sugars to determine whether there are additive effects of combining high levels of both these macronutrients compared to their individual effects on offspring health. Therefore, the relative contribution of specific macronutrients in the diet to the development of the metabolic syndrome in the offspring remains unclear.

## 6. Resveratrol: A Nutritional Intervention That Prevents the Deleterious Effects of Excess Macronutrients in the Maternal Diet on the Offspring?

It is generally accepted that good nutritional practice combined with maintaining physical activity can prevent many chronic diseases. However, in pregnant women increasing caloric intake is necessary in order to provide nutrition for the growing infant. It is unlikely that eating less and starting to become physically active during pregnancy for the purpose of creating a desirable metabolic profile will gain widespread acceptance. Therefore, in rodent model systems, the administration of natural molecules present in plant-based foods are being explored as possible therapeutics to prevent the adverse effects of maternal over-nutrition on the offspring. Among these, resveratrol which is a polyphenolic molecule that is naturally produced by plants in response to environmental pathogens, is the most widely studied [97]. Resveratrol is present in many types of vegetables, fruits, and nuts, including peanuts, berries, grapes, and red wine, although most nutritional supplements of resveratrol are produced from Japanese knotweed, which has a very high content [98]. Resveratrol mimics several of the biological effects of restricting macronutrient calories in the absence of micronutrient deficiency [99]. Resveratrol has been found to have many pleiotropic effects which, at least in animal model systems, improves health in various disease models including cancer, diabetes, cardiovascular and Alzheimer’s diseases, indicating that resveratrol could function as a broad-spectrum therapeutic agent for varied chronic diseases that appear to have a developmental origin (as reviewed in [100]). For example, in diabetic rats and nonhuman primates, resveratrol lowered blood glucose levels through both insulin dependent and independent pathways and has been found to promote anti-inflammatory and anti-apoptotic responses [101,102,103]. It is commonly believed that resveratrol primarily works by activating the 5′ AMP-activated protein kinase (AMPK), which is required for proper glucose homeostasis and mitochondrial fatty acid oxidation [102,104]. Resveratrol exists in both *cis* and *trans* isomers, however, the *trans* form is a more effective antioxidant, more biologically active, and more stable [97].

Maternal resveratrol supplementation of high-calorie diets during pregnancy has been found to have some benefits for maternal metabolic health in several animal studies. For example, in pregnant Japanese macaques fed a 36% saturated fat diet, supplementation with 0.37% resveratrol for three months before breeding and during pregnancy induced increased maternal weight loss, improved glucose tolerance and uterine artery blood flow [103]. In another study, pregnant *db*/+ mice fed a diet consisting of 47% carbohydrate and 17% fat supplemented with 10 mg/kg/day of resveratrol had lower maternal circulating glucose levels and improved insulin tolerance [105]. Notably, pregnant streptozotocin-induced diabetic Sprague–Dawley rats supplemented with 100 mg/kg resveratrol in their diet had reduced levels of cholesterol, triglycerides, and glucose in their circulation and an up-regulation in their anti-oxidant production [104].

In addition to the improvements in maternal metabolic health, resveratrol supplementation during pregnancy was observed to be safe, non-teratogenic and well tolerated by the fetus [104,106,107]. In fact, in a rat model of severe hypoxia during pregnancy, resveratrol prevented small for gestational age offspring [106]. Yao et al. [105] found that resveratrol supplementation of high-fat diets of pregnant *db*/+ mice increased fetal survivability and lowered offspring body weights. They argue that AMPK was up-regulated in the livers, which in turn lowered glucose-6-phosphatase expression that led to a reduction in gluconeogenesis and a reduction in glucose output [105]. Additionally, Zou et al. [108] found that 0.2% *w*/*w* resveratrol supplementation in pregnant C57BL/6 J mice consuming a high-fat diet (45% energy from fat) promoted brown and beige adipocyte development in white adipose tissue, which is important for increased thermogenesis and energy expenditure, in the male offspring. Furthermore, the male offspring from the dams that consumed a high-fat diet supplemented with resveratrol exhibited better insulin sensitivity and were less obese than the offspring from the high-fat diet dams without supplementation, thus protecting these offspring from metabolic disorder development [108]. Interestingly, one study found that the fetal pancreas mass was increased by 42%, and though fetal islet mass and β-cell mass were not affected and insulin gene expression was reduced [103]. On the other hand, α-cell mass in the offspring was reduced by maternal resveratrol supplementation, though glucagon gene expression was increased [103]. However, functional assays to assess the effects of maternal resveratrol supplementation on insulin and glucagon secretion by the islets from the offspring were not performed in this study and given the improved glucose tolerance in the dams it is difficult to conclude that maternal resveratrol had adverse effects on β-cell function and glycemic control in the Japanese macaque, suggesting further mechanistic studies of resveratrol and pancreatic islet function are needed. These authors did observe that Ki67 expression, a proliferation marker, and Bcl2, an anti-apoptotic marker, were increased throughout the exocrine pancreas of the offspring of resveratrol-fed dams during the third trimester, suggesting that the pancreas was in a highly proliferative state [103], which explains why pancreas mass was increased. Interestingly this is opposed to the body of literature that suggests that resveratrol has anti-proliferative effects on adult cells (as reviewed in [109]). All of these findings suggest that resveratrol supplementation in maternal diets during pregnancy could potentially improve metabolic health outcomes in both mothers and their offspring; however, more research should be conducted to ensure the safety of resveratrol and, specifically for pancreas development in the offspring.

## 7. Future Perspectives

The studies we reviewed focused on elucidating the effects of altered maternal macronutrient composition on the long-term health of the offspring. Animal model studies provide strong evidence that the consumption of diets that are high in saturated fats and/or simple sugars during pregnancy or lactation promote obesity and a range of metabolic and cardiovascular disorders in the offspring and shed new light on possible mechanisms that are involved. A limitation of this approach is the difficulty in separating the effects of macronutrient excess from gestational weight gain, which precludes definitive conclusions about which specific maternal factor is most influential on offspring health. Excessive gestational weight gain has been associated with an increased risk for obesity and other metabolic conditions that are independent of current lifestyle factors in the offspring. However, in a very elegant study, the presence of maternal insulin resistance in the absence of maternal obesity and excessive gestational weight gain appeared to be sufficient to induce metabolic dysfunction in the offspring. Using pregnant insulin receptor substrate-1 heterozygous null mice that are insulin resistant, Isganaitis et al. showed that their wild-type male offspring were glucose intolerant, developed hepatic steatosis and were more sensitive to high-fat diet-induced obesity than their respective wild-type control male offspring of insulin sensitive wild-type dams [110].

There is also a lack of studies that characterize how high levels of certain fats or simple carbohydrates in the maternal diet directly affect the fetus. Observational studies in humans suggest that maternal obesity is associated with increased amino acid and glucose transporter expression in the placenta [111]. In mice, high-fat diet feeding during pregnancy increased placental amino acid and glucose transporter 1 expression in conjunction with heavier mouse offspring [112]. These observations suggest that macronutrient excess has a direct effect on placental nutrient transport that ultimately affects fetal growth, development and postnatal health.

Studies are only beginning to emerge examining how maternal macronutrient composition affects epigenetic mechanisms. Future research in this field should also take advantage of metabolomic, proteomic, transcriptomic and epigenomic technologies to gain a more integrated view of the complex effects of how altered maternal macronutrient diet compositions affect the developmental origins of disease in metabolically active tissues. The controlled dietary conditions provided by animal model research will assist in ultimately identifying how suboptimal nutrition, in particular, maternal dietary macronutrient components, affect the tissue level mechanisms that control gene expression and condition the offspring for metabolic disorders. Understanding the mechanisms by which the macronutrient composition of the maternal diet during pregnancy or lactation affects health outcomes in the offspring could lead to improved maternal dietary recommendations and better disease prevention strategies and therapies for the offspring.

## Figures and Tables

**Table 1:** Summary of evidence for long-term influences of maternal low-protein diets on offspring health outcomes.

Protein %	Diet Protocol	Findings	Reference
50% or 100% of total nutrient requirements. Diet provided 9.6 MJ/kg (metabolizable energy—megajoules/kilogram) and 14.75 g of crude protein.	Female ewes (second or third pregnancy) were fed control or restricted nutrient diets between days 1–31 of gestation and 100% of nutrient requirements after day 31, during delivery and lactation, until lambs were weaned at 12 weeks of age. Offspring fed ad libitum or to a level that reduced body weight to 85% of individual target weight from 12–25 weeks of age. All offspring received 100% of nutritional requirements from 25 weeks of age onwards.	Cardiac hypertrophy altered cardiac function in male offspring protein restricted during pregnancy; high blood pressure in male offspring protein restricted in early postnatal life.	Cleal et al. 2007 [30]
Isocaloric low-protein diet (8% protein) vs. control diet (20% protein).	C57/b16 mice fed during gestation and lactation. Offspring cross-fostered to control (born and suckled by control diet dams), postnatal low-protein (born to control dams, suckled by low-protein dams) and recuperated (born to low-protein dams, sucked by control dams; three experimental groups) until postnatal day 21.	Improved insulin sensitivity in offspring exposed to protein restriction throughout pregnancy and lactation (increased PKC-ζ expression). Impaired insulin resistance in offspring who were protein restricted during pregnancy only.	Chen et al. 2009 [25]
Isoenergetic low-protein (8% protein *wt*/*vol*) vs. control diet (20% protein *wt*/*vol*).	Wistar Han rats fed during gestation and lactation. Offspring were weaned onto standard chow at postnatal day 21 until 14 months of age.	Decreased adipocyte size, impaired insulin sensitivity of adipocytes, reduced Akt expression.	Martin-Gronert et al. 2016 [29]
Isocaloric low-protein (8% protein) vs. control diet (20% protein). Maternal low-protein diet supplemented with carbohydrate to match the calorie content of control diet.	Pregnant ApoE^−/−^ mice (C57BL6/J) fed during pregnancy and lactation. Offspring weaned onto standard chow containing 20% protein at postnatal day 21 until six months of age.	Increased atherosclerotic plaque in aorta of male offspring, elevated LDL-cholesterol levels, increased fasting insulin levels, increased HMG-CoA reductase levels in liver.	Blackmore et al. 2012 [31]
Low protein (8% casein) vs. normal protein (17% protein). Both diets were isoenergetic, low-protein diet differed from normal protein diet in the content of carbohydrate and protein.	Female Wistar rats were fed standard chow (52% carbohydrate, 21% protein, 4% lipids) until confirmation of pregnancy, when they were switched to the experimental diets for the duration of pregnancy and lactation. Offspring received standard chow at weaning.	Increased blood pressure, fasting insulin levels, blood lipid levels.	de Brito Alves et al. 2016 [23]
Low-protein diet (6% protein) vs. control diet (20% protein). Both diets were isocaloric (3.8 kcal/g), but differed in the amount of protein (casein and dl-Methionine).	C57BL/6J female mice fed experimental diets for two weeks after which they were bred. Fetuses and placentas were dissected at embryonic days 10.5, 17.5 and 18.5.	Altered placental function.	Gonzalez et al. 2016 [20]
Isocaloric low-protein (8% *wt*/*vol* protein) vs. control diet (20% *wt*/*vol* protein).	Female Wistar rats fed experimental diets upon confirmation of pregnancy, throughout gestation and lactation. Offspring weaned onto standard rat diet at postnatal day 21 for the remainder of the study.	Increased blood pressure, fasting insulin levels, blood lipid levels.	Fernandez-Twinn et al. 2005 [22]
Restricted isocaloric diet (10% casein) vs. control diet (20% casein).	Female Wistar rats fed experimental diets upon confirmation of pregnancy, throughout gestation and lactation. Offspring were cross-fostered producing four experimental groups: control (from dams receiving control diet during pregnancy and lactation), restricted (from dams receiving restricted diet during pregnancy and lactation), control-restricted and restricted-control. On postnatal day 21 all pups were weaned onto control diet.	Improved insulin sensitivity in male and female offspring exposed to protein restriction throughout pregnancy and lactation or lactation only.	Zambrano et al. 2006 [24]
Low protein (8% protein) vs. standard protein (19% protein). Diets were isoenergetic.	Female Sprague–Dawley rats fed experimental diets upon confirmation of pregnancy, throughout gestation and lactation. Offspring were weaned onto standard laboratory chow on postnatal day 28, remaining on the diet for the duration of the study.	Decreased hepatic triacylglycerol content in male offspring from protein restricted dams. Mediated through increased fatty acid transport to the mitochondria or altered biosynthesis.	Qasem et al. 2010 [26], Qasem et al. 2015 [27]
Isoenergetic corn–barley, soybean meal diets (13.7 MJ of ME/kg) containing high (30%, 1:1.3 protein:carbohydrate ratios), low (6.5%, 1:10.4 (protein:carbohydrate ratios) or adequate (12.1%, 1:5 protein:carbohydrate ratios) protein diets.	Female pigs were bred by artificial insemination and randomly assigned to dietary treatments which were continued throughout gestation. Piglets were cross-fostered to female pigs fed a standard diet during pregnancy and lactation. Piglets had access to standard diet two weeks before weaning and for the remainder of the study.	Reduced lean mass, increased fat mass, reduced muscle myofibers and reduced *IGF-2* mRNA expression	Rehfeldt et al. 2012 [28]
Isocaloric low-protein (8%) diet vs. control protein (20%) diet.	Pregnant Wistar rats were maintained on experimental diets throughout pregnancy and lactation. Pups born to low-protein diet dams were cross-fostered to control fed mothers. Pups born to control diet dams were suckled by control diet dams. On postnatal day 21 pups were weaned onto standard laboratory chow or standard laboratory chow supplemented with CoQ10 (1 mg/kg body weight per day) and maintained on their respective diets until 12 months of age.	Accelerated catch-up growth following exposure to maternal protein restriction, increased hepatic fibrosis, inflammation and lipid peroxidation	Tarry-Adkins et al. 2016 [32]
Low-protein diet (8.7% casein) vs. normal protein diet (20% casein). Nutrient content of the diets was equivalent (vitamins, minerals, methionine, oils), except for starch, which was altered to ensure the diets were isocaloric.	Wistar Kyoto rat dams fed experimental diets two weeks before mating, during pregnancy and for two weeks after giving birth. Offspring were weaned onto standard chow until 32 weeks of age.	Growth-restricted male and female offspring maintained throughout study (i.e., no catch-up growth), increased insulin sensitivity in protein-restricted offspring	Lim et al. 2011 [33]

**Table 2:** Summary of evidence for long-term influences of maternal high-sugar diets on offspring health outcomes.

Sugar %	Experimental Findings	Reference
75% vs. 35% Dextrose and maltodextrin	Higher body weights in male offspring.	Shankar et al., 2008 [48] & 2010 [49] & 2010 [50] Borengasser et al., 2014 [51] & 2011 [52] & 2013 [53]
	Increased obesity and percent body fat in male offspring.	Shankar et al., 2008 [48]
	Upregulation of lipogenic and adipogenic genes in white adipose tissue due to changes in DNA methylation in *PPAR-γ*, *CCAAT enhancer binding protein-α* and *-β* leading to increased obesity in male offspring.	Borengasser et al., 2013 [53]
	Hyperinsulinemia, hyperleptinemia, increased resistin levels, leading to insulin resistance in male offspring.	Shankar et al., 2010 [49]
	Both diets resulted in hyperglycemia, increased triglycerides, insulin and leptin levels in serum of male offspring. Negative response to an oral glucose tolerance test (insulin intolerant) in male offspring.	Shankar et al., 2008 [48]
	Downregulation of hepatic mitochondrial function markers (*SIRT3*, mitochondrial protein content, electron transport chain complexes II, III, *ATPase*, and PGC-1α mRNA) of male offspring.	Borengasser et al., 2011 [52]
	Downregulation of mitochondrial factors required for proper fusion and fission (*PARL*, *optic atrophy 1*, *mitofusin 1* and *2*, *fission 1*, *nuclear respiratory factor 1*) in the liver of male offspring.	Borengasser et al., 2014 [51]
50% Fructose	Hyperglycemia in both male and female pups.	Jen et al., 1991 [54]
60% Fructose	Hyperinsulinemia, elevated serum lipids. Changes in lipid metabolism genes (increased *acetyl-CoA carboxylase-2*, *CPT-1*α, reduced *PPAR-α* and *PGC-1*α) in the livers of male offspring. Hypertension, downregulation in genes controlling blood pressure in the kidneys of male offspring.	Ching et al., 2011 [55] Tain et al., 2015 [56]
10% Fructose	Impaired fetal leptin signaling, increased body weight and food consumption.	Rodriguez et al., 2013 [57] & Alzamendi et al., 2010 [58]
20% Fructose	Alterations in neonatal liver lipid metabolism, no obesity observed, increased liver triglycerides and increased molecular markers of ER stress. Male offspring had a reduction in genes involved in free fatty acid metabolism in the liver (*ACAT1*, *Acsl4*, *Acad10*, *and CPT-1*α). Female offspring were not affected.	Clayton et al., 2015 [59]
20% Sucrose	Increased angiotensin II in blood, increased vasoconstriction in aorta and mesenteric arteries of male offspring.	Wu et al., 2016 [60]

**Table 3:** Summary of evidence for long-term influences of maternal high fat on offspring health outcomes.

Fat %	Diet Protocol	Findings	Reference
Standard chow with 20% *w*/*w* animal lard vs. standard chow (5% *w*/*w* fat).	Female SD rats fed for 10 days prior to mating, throughout pregnancy.	No alteration in uterine artery function	Taylor et al. 2003 [64]
High-fat diet (60% kcal from fat, 20% protein, 20% carbohydrate, 5.24 kcal/g energy) vs. standard chow (17% kcal from fat, 25% protein, 58% carbohydrate, 3.1 kcal/g energy).	C57BL6J mice fed starting on gestational day 1.	Increased adiposity at E18.5, elevated free fatty acid levels at E18.5	Qiao et al. 2015 [65]
Standard chow with 20% *w*/*w* animal lard vs. standard chow (5% *w*/*w* fat).	Female SD rats fed for 10 days prior to mating, throughout pregnancy and lactation. Offspring weaned onto standard chow.	Insulin resistance, impaired glucose-stimulated insulin secretion, lower mitochondrial DNA copy number	Taylor et al. 2005 [66]
Standard chow (13.5% kcal from fat) vs. high-fat diet (60% kcal from fat).	Pregnant SD rats fed starting on gestational day 2, throughout pregnancy and lactation. Offspring were cross-fostered to standard chow or high-fat dams on P1 (four experimental groups). Offspring weaned onto standard chow.	Increased adiposity and body weight in male offspring	Sun et al. 2012 [67]
Control diet supplemented *w*/*w* with animal lard, (25.7% fat, 19.5% protein, 41.3% carbohydrates and 3.5% fiber) vs. control diet (5.3% fat from corn oil, 21.2% protein, 57.4% carbohydrates and 4.6% fiber). The greatest composition of fat in the high-fat diet was estimated to be oleic acid, palmitic acid, and stearic acid.	Female SD rats fed for 10 days prior to mating, throughout pregnancy and lactation. Offspring weaned onto standard chow.	Alterations in endothelial function, hypertension in female offspring, no change in lipid profile	Khan et al. 2003 [68]
Control diet supplemented *w*/*w* with animal lard, (25.7% fat, 19.5% protein, 41.3% carbohydrates and 3.5% fiber) vs. control diet (5.3% fat from corn oil, 21.2% protein, 57.4% carbohydrates and 4.6% fiber). The greatest composition of fat in the high-fat diet was estimated to be oleic acid, palmitic acid, and stearic acid.	Female SD rats fed for 10 days prior to mating, throughout pregnancy and lactation. Offspring from high-fat-fed dams were weaned onto standard chow or high-fat diet. Offspring from control fed dams were weaned onto standard chow.	Alterations in endothelial function, hypertension in female offspring, no change in lipid profile	Khan et al. 2004 [2]
Control diet supplemented *w*/*w* with animal lard, (25.7% fat, 19.5% protein, 41.3% carbohydrates and 3.5% fiber) vs. control diet (5.3% fat from corn oil, 21.2% protein, 57.4% carbohydrates and 4.6% fiber). The greatest composition of fat in the high-fat diet was estimated to be oleic acid, palmitic acid, and stearic acid.	Female SD rats fed for 10 days prior to mating, throughout pregnancy and lactation. Offspring were cross-fostered to standard chow or high-fat-fed dams on P1 (four experimental groups). Offspring weaned onto standard chow.	Increased male offspring body weight, hypertension in female offspring, no change in lipid profile	Khan et al. 2005 [69]
High omega-6 polyunsaturated fat diet (59% fat from safflower oil, 21% protein, 20% carbohydrate) vs. standard chow (12% fat, 23% protein, 65% carbohydrate).	Female Wistar rats fed for four weeks prior to mating and throughout pregnancy. During lactation, all dams were fed standard chow. Offspring weaned onto standard chow.	Increased body fat:lean mass ratio in offspring exposed to omega-6 rich diet, reduced IR-β, IRS expression in liver, increased PKC-ζ expression	Buckley et al. 2005 [70]

**Table 4:** Summary of evidence for long-term influences of maternal high fat and sucrose on offspring health outcomes.

Fat %	Diet Protocol	Findings	Reference
High-fat diet (45% kcals from fat, D12451, Research Diets) vs. standard chow	Female Wistar rats fed from P22 to P120. Three dietary groups were established during pregnancy: 1. controls − fed standard chow throughout their life, pregnancy and lactation; 2. maternal high fat − fed high-fat diet throughout their life, pregnancy and lactation; 3. pregnancy + lactation high fat − fed standard chow throughout their life and fed high-fat diet during pregnancy and lactation only. Offspring weaned onto standard chow or high-fat diet.	Microsomia at birth, obesity at 5 months of age in maternal HF and pregnancy + lactation HF groups Hyperinsulinemia Hyperleptinemia (correlated to fat mass)	Howie et al. 2009 [74]
High-fat diet (45% kcals from fat, D12451, Research Diets) vs. standard chow (18% kcals from fat)	Female Wistar rats fed diets at the start of pregnancy and throughout lactation. Offspring weaned onto standard chow.	Microsomia at birth followed by catch-up growth at P2 Altered cell cycle dynamics in P2 offspring livers (hypomethylated *Cdkn1α*)	Dudley et al. 2011 [75]
High-fat diet (45% kcal fat, 20% kcal protein, 35% kcal carbohydrate) vs. standard chow (21% kcal fat, 17% kcal protein, 63% kcal carbohydrate)	Female C57BL6J mice fed four weeks before mating, throughout pregnancy and lactation. Offspring weaned onto control or high-fat diets (four experimental groups).	Obesity, liver steatosis (NAFLD) and liver inflammation, elevated levels of gene expression associated with oxidative stress, inflammation, de novo lipogenesis	Bruce et al. 2009 [76]
High-fat + sucrose diet (45% kcal fat, Research Diets D12451) vs. Low-fat diet (10% kcal fat, Research Diets D12450B)	Female SD rats six weeks prior to mating, throughout pregnancy and lactation. Offspring weaned onto low fat or high-fat + sucrose diets (four experimental groups).	Obesity, hepatic steatosis, insulin resistance, altered hepatic metabolome, reduced gene expression of *Pcyt2* and *PPAR-α* (key regulators of hepatic lipid metabolism)	Pereira et al. 2015 [45]
High fat + sucrose diet (45% kcal fat, Research Diets D12451) vs. Low-fat diet (10% kcal fat, Research Diets D12450B)	Female SD rats six weeks prior to mating, throughout pregnancy and lactation. Offspring weaned onto low fat or high-fat + sucrose diets (four experimental groups.)	Sustained elevation of IL-1β and IL-10 levels in spleen cells upon stimulation of TLR), IL-1β positively correlated with maternal body weight, glucose, free fatty acid, and triglyceride levels	Li et al. 2016 [77]
Obesogenic diet (10% simple sugars, 20% animal lard, 28% polysaccharide, 23% protein (*w*/*w*), 4.5 kcal/g energy) supplemented with sweetened condensed milk (55% simple sugars, 8% fat, 8% protein (*w*/*w*)) vs. standard chow (7% simple sugars, 3% fat, 50% polysaccharide, 15% protein (*w*/*w*), 3.5 kcal/g energy). Macronutrient intake for the obesogenic group was estimated to be 16% fat, 33% simple sugars, 15% protein, 4.0 kcal/g energy	Female C58BL6J mice (proven breeders) fed for six weeks prior to mating, throughout pregnancy and lactation. Offspring weaned onto standard chow.	Elevated systolic and MAP in male & female offspring, increased pancreatic insulin content, elevated *PPAR-γ* gene expression	Samuelsson et al. 2008 [78]
Obesogenic diet (10% simple sugars, 20% animal lard, 28% polysaccharide, 23% protein(*w*/*w*), 4.5 kcal/g energy) supplemented sweetened condensed milk (55% simple sugars, 8% fat, 8% protein (*w*/*w*)) vs. standard chow (7% simple sugars, 3% fat, 50% polysaccharide, 15% protein(*w*/*w*), 3.5 kcal/g energy). Macronutrient intake for the obesogenic group was estimated to be 16% fat, 31% simple sugars, 28% polysaccharides, 18% protein, 7% other and 4% fat, 6% simple sugars, 46% polysaccharides, 22% protein, 22% other for the control group	Female SD rats fed six weeks prior to mating, throughout pregnancy and lactation. Offspring weaned onto standard chow.	Increased adiposity and hyperphagia, elevated leptin gene expression in adipose tissue	Kirk et al. 2009 [79]
Obesogenic diet (10% simple sugars, 20% animal fat(*wt*/*wt*)) supplemented with sweetened condensed milk (55% simple sugars, 8% fat, 8% protein(*wt*/*wt*)) vs. standard chow (7% simple sugars, 3% fat(*wt*/*wt*))	Female C57BL6J mice fed six weeks prior to first pregnancy (to determine if proven breeders), throughout second pregnancy and lactation. Offspring weaned onto standard chow.	Cardiac hypertrophy (morphometric and molecular markers) hyperinsulinemia, increased oxidative stress	Fernandez-Twinn et al. 2012 [80]
Obesogenic diet (10% simple sugars, 20% animal lard, 28% polysaccharide, 23% protein(*w*/*w*), 4.5 kcal/g energy) supplemented with sweetened condensed milk (55% simple sugars, 8% fat, 8% protein(*w*/*w*)) vs. standard chow (7% simple sugars, 3% fat, 50% polysaccharide, 15% protein (*w*/*w*), 3.5 kcal/g energy)	Female C57BL/6J mice (proven breeders) fed for six weeks prior to second mating, throughout pregnancy and lactation. Offspring were weaned onto standard chow.	Decreased insulin signaling expression in female skeletal muscle, decreased mitochondrial complex expression in male skeletal muscle	Shelley et al. 2009 [81]
Obesogenic diet (10% simple sugars, 20% animal fat (*w*/*w*)) supplemented with sweetened condensed milk (55% simple sugars, 8% fat, 8% protein (*w*/*w*)) vs. standard chow (7% simple sugars, 3% fat (*w*/*w*))	Female C57BL6J mice fed six weeks prior to first pregnancy (to determine if proven breeders), throughout second pregnancy and lactation. Offspring weaned onto standard chow.	Elevated serum insulin levels, downregulated insulin signaling pathway in adipose tissue	Fernandez-Twinn et al. 2014 [82]
Obesogenic diet (10% simple sugars, 20% animal lard, 28% polysaccharides, 23% protein (*wt*/*wt*), 28.43 kJ/g) supplemented with sweetened condensed milk (16% fat, 33% simple sugars, 15% protein, 13.7 kJ/g) vs. standard chow (7% simple sugars, 3% fat, 50% polysaccharide, 15% protein (*wt*/*wt*), 10.74 kJ/g)	Female C57BL6J mice fed six weeks prior to first pregnancy (to determine if proven breeders), throughout second pregnancy and lactation. Offspring weaned onto standard chow.	Hyperinsulinemia, markers of oxidative damage and mitochondrial dysfunction in liver, increased hepatic lipid accumulation (NAFLD)	Alfaradhi et al. 2014 [83]
Obesogenic diet (10% simple sugars, 20% animal lard (*wt*/*wt*)) supplemented with sweetened condensed milk (55% simple sugars, 8% fat) vs. standard chow (7% simple sugars, 3% fat (*wt*/*wt*))	Female C57BL6J mice fed six weeks prior to first pregnancy (to determine if proven breeders), throughout second pregnancy and lactation. Offspring weaned onto standard chow.	Cardiac hypertrophy (re-expression of fetal gene program), systolic and diastolic cardiac dysfunction	Blackmore et al. 2014 [84]
Obesogenic diet (6.79 kcal/g, 45% kcal fat) supplemented with sweetened condensed milk (55% simple sugars, 8% fat, 8% protein (*w*/*w*)) vs. standard chow (2.56 kcal/g, 7.42% kcal fat)	Female C57BL6J mice fed six weeks prior to first pregnancy (to determine if proven breeders), throughout second pregnancy and lactation. Offspring weaned onto standard chow.	Adipose tissue cytokine and chemokine signaling elevated	Alfaradhi et al. 2016 [85]
Obesogenic diet (10% simple sugars, 29% polysaccharide, 23% fat (17% animal lard), 23% protein (*wt*/*wt*), 18.83 kJ/g energy) supplemented with sweetened condensed milk (55% simple sugars, 8% fat, 8% protein (*wt*/*wt*), 16.736 kJ/g energy) vs. control chow (7% simple sugars, 50% polysaccharide, 3% fat, 15% protein (*wt*/*wt*), 14.64 kJ/g energy)	Female Wistar rats fed 60 days prior to mating, throughout pregnancy and lactation. Offspring weaned onto control diet.	Hyperphagia, obesity, insulin resistance	Nivoit et al. 2009 [86]
Obesogenic diet (10% simple sugars, 18% animal lard, 4% soya oil, 28% polysaccharide, 23% protein (*w*/*w*), 4.5 kcal/g energy) supplemented with sweetened condensed milk (55% simple sugars, 8% fat, 8% protein (*w*/*w*)) vs. standard chow (7% simple sugars, 3% fat, 50% polysaccharide, 15% protein (*w*/*w*), 3.5 kcal/g energy)	Female C57BL/6J mice (proven breeders) fed for six weeks prior to second mating, throughout pregnancy and lactation. Offspring weaned onto standard chow or obesogenic diet.	Hepatic steatosis and fibrosis (NAFLD), hepatic inflammation, elevated hepatic triglyceride levels	Mouralidarane et al. 2013 [87]
Obesogenic diet (10% simple sugars, 20% animal lard, 28% polysaccharide, 23% protein (*w*/*w*), 4.5 kcal/g energy) supplemented with sweetened condensed milk (55% simple sugars, 8% fat, 8% protein (*w*/*w*)) vs. standard chow (7% simple sugars, 3% fat, 50% polysaccharide, 15% protein (*w*/*w*), 3.5 kcal/g energy). Macronutrient intake for the obesogenic diet was calculated to be 16% fat, 33% simple sugars, 15% protein and 4.0 kcal/g energy	Female C57BL/6J mice (proven breeders) were fed six weeks prior to mating, throughout pregnancy and lactation. A subgroup of offspring was weaned onto standard chow or obesogenic diet. A subgroup of offspring was cross-fostered to an obesogenic or control fed dam and weaned onto standard chow.	Body weight gain, insulin resistance, NAFLD	Oben et al. 2010 [88]
Obesogenic diet (10% simple sugars, 18% animal lard, 4% soya oil, 28% polysaccharide, 23% protein (*w*/*w*), 4.5 kcal/g energy) supplemented with sweetened condensed milk (55% simple sugars, 8% fat, 8% protein (*w*/*w*)) vs. standard chow (7% simple sugars, 3% fat, 50% polysaccharide, 15% protein (*w*/*w*), 3.5 kcal/g energy). Macronutrient intake for the obesogenic diet was calculated to be 16% fat, 33% simple sugars, 15% protein and 4.0 kcal/g energy	Female C57BL/6J mice were fed six weeks before mating, throughout pregnancy and lactation. Offspring weaned onto the control diet or obesogenic diet (four experimental groups).	Body weight gain, altered pro-apoptotic and autophagy markers in the pancreas	Soeda et al. 2016 [89]
